# Phase IB study of doxorubicin in combination with the multidrug resistance reversing agent S9788 in advanced colorectal and renal cell cancer.

**DOI:** 10.1038/bjc.1997.563

**Published:** 1997

**Authors:** C. J. Punt, E. E. Voest, E. Tueni, A. T. Van Oosterom, A. Backx, P. H. De Mulder, B. Hecquet, C. Lucas, B. Gerard, H. Bleiberg

**Affiliations:** Department of Medical Oncology, University Hospital Nijmegen, The Netherlands.

## Abstract

S9788 is a new triazineaminopiperidine derivate capable of reversing multidrug resistance (MDR) in cells resistant to chemotherapeutic agents such as doxorubicin. It does not belong to a known class of MDR revertants, but its action involves the binding of P-glycoprotein. Thirty-eight evaluable patients with advanced colorectal or renal cell cancer were treated with doxorubicin alone (16 patients) followed after disease progression with combination treatment of doxorubicin plus S9788 (12 patients) or upfront with the combination of doxorubicin plus S9788 (22 patients). S9788 was given i.v. as a loading dose of 56 mg m-2 over 30 min followed by doxorubicin given at 50 mg m-2 as a bolus infusion. Thereafter, a 2-h infusion of S9788 was administered at escalating doses ranging from 24 to 120 mg m-2 in subsequent cohorts of 4-10 patients. Pharmacokinetic analysis demonstrated that concentrations of S9788 that are known to reverse MDR in vitro were achieved in patients at non-toxic doses. Compared with treatment with doxorubicin alone, treatment with the combination of doxorubicin and S9788 produced a significant increase in the occurrence of WHO grade 3-4 granulocytopenia. Treatment with S9788 was cardiotoxic as it caused a dose-dependent and reversible increase in corrected QT intervals as well as clinically non-significant arrhythmias on 24- or 48-h Holter recordings. Although clinically relevant cardiac toxicities did not occur, the study was terminated as higher doses of S9788 may increase the risk of severe cardiac arrhythmias. Twenty-nine patients treated with S9788 plus doxorubicin were evaluable for response, and one patient, who progressed after treatment with doxorubicin alone, achieved a partial response. We conclude that S9788 administered at the doses and schedule used in this study results in relevant plasma concentrations in humans and can safely be administered in combination with doxorubicin.


British Journal of Cancer (1997) 76(10), 1376–1381
© 1997 Cancer Research Campaign

### Phase IB study of doxorubicin in combination with the multidrug resistance reversing agent S9788 in advanced colorectal and renal cell cancer

**CJA Punt[1], EE Voest[3], E Tueni[4], AT Van Oosterom[5], A Backx[2], PHM De Mulder[1], B Hecquet[6], C Lucas[7], B Gerard[7] and H Bleiberg[8].**

Departments of [1]Medical Oncology and [2]Cardiology, University Hospital Nijmegen, The Netherlands; [3]Department of Internal Medicine, University Hospital Utrecht, The Netherlands; [4]Jolimont Hospital, Tivoli, Belgium; [5]Department of Medical Oncology, University Hospital Antwerp, Belgium; [6]Center Oscar Lambret, Lille, France; [7]IRI Servier, Courbevoie, France; [8]Institute Jules Bordet, Brussels, Belgium

**Summary** S9788 is a new triazineaminopiperidine derivate capable of reversing multidrug resistance (MDR) in cells resistant to chemotherapeutic agents such as doxorubicin. It does not belong to a known class of MDR revertants, but its action involves the binding of P-glycoprotein. Thirty-eight evaluable patients with advanced colorectal or renal cell cancer were treated with doxorubicin alone (16 patients) followed after disease progression with combination treatment of doxorubicin plus S9788 (12 patients) or upfront with the combination of doxorubicin plus S9788 (22 patients). S9788 was given i.v. as a loading dose of 56 mg $m^{-2}$ over 30 min followed by doxorubicin given at 50 mg $m^{-2}$ as a bolus infusion. Thereafter, a 2-h infusion of S9788 was administered at escalating doses ranging from 24 to 120 mg $m^{-2}$ in subsequent cohorts of 4–10 patients. Pharmacokinetic analysis demonstrated that concentrations of S9788 that are known to reverse MDR in vitro were achieved in patients at non-toxic doses. Compared with treatment with doxorubicin alone, treatment with the combination of doxorubicin and S9788 produced a significant increase in the occurrence of WHO grade 3–4 granulocytopenia. Treatment with S9788 was cardiotoxic as it caused a dose-dependent and reversible increase in corrected QT intervals as well as clinically non-significant arrhythmias on 24- or 48-h Holter recordings. Although clinically relevant cardiac toxicities did not occur, the study was terminated as higher doses of S9788 may increase the risk of severe cardiac arrhythmias. Twenty-nine patients treated with S9788 plus doxorubicin were evaluable for response, and one patient, who progressed after treatment with doxorubicin alone, achieved a partial response. We conclude that S9788 administered at the doses and schedule used in this study results in relevant plasma concentrations in humans and can safely be administered in combination with doxorubicin.

Inherent or acquired multidrug resistance (MDR) is an important cause of failure of cancer chemotherapy. Several mechanisms responsible for MDR have been described, and the most extensively investigated is the expression of the product of the *MDR1* gene, P-glycoprotein (P-gp) or P-170 (Roninson, 1992). This protein acts as an efflux pump for a number of commonly used cytotoxic agents, e.g. doxorubicin, vincristine, vinblastine and actinomycin D. Different compounds have been shown to reverse P-gp-mediated MDR, including calcium channel antagonists (verapamil), calmodulin inhibitors (quinine, quinidine), oestrogen receptor antagonists (tamoxifen), steroids and immunomodulators (cyclosporin A). The mechanisms by which these drugs influence MDR have not always been identified. The clinical use of these MDR modulators is hampered by the toxic side-effects that occur when the suprapharmacological doses required to achieve significant reversal of MDR are used (Pennock et al, 1991; Raderer and Scheithauer, 1993). Therefore, the search for novel and more potent MDR modulators is of major importance.

S9788 is a triazineaminopiperidine derivate capable of reversing MDR in vitro and in vivo in a dose-dependent way (Cros et al, 1992; Pierre et al, 1992). Structurally, S9788 does not belong to any of the classes of compounds known to reverse MDR. It increases the intracellular accumulation and retention of doxorubicin, vincristine and vinblastine in resistant cell lines displaying the P-gp-mediated MDR phenotype (Leonce et al, 1992; Efferth et al, 1993; Hill et al, 1993; Huet et al, 1993; Julia et al, 1994; Merlin et al 1994). These studies also showed that modulation of resistance is obtained in both drug-selected and inherently resistant cell lines, and that S9788 is more potent than verapamil at equimolar concentrations. It has been hypothesized that, in addition to P-gp binding, S9788 may also act by altering the intracellular drug distribution (Merlin et al, 1994; Sebille et al, 1994). Although the optimum schedule has not yet been defined, a prolonged infusion of S9788 starting before and maintained after the administration of chemotherapy appears to be more effective than a single-bolus infusion (Perez et al, 1993; Julia et al, 1994; Soudon et al, 1995). S9788 has been shown not to interfere with doxorubicin plasma pharmacokinetics (de Valeriola et al, 1997). Preliminary results of phase I studies of doxorubicin plus S9788, the latter being infused over 30 min or 6 h, have shown cardiac arrhythmias (mainly AV blocks, bradycardia, tachycardia), hypotension and prolongation of the QT interval on electrocardiograms in some patients (Awada et al, 1993; Clavel et al, 1993; Goncalves et al, 1995). Most of these cardiac toxicities were asymptomatic. This prompted us to keep patients under close cardiac surveillance in the present study. Only patients with

*Received 17 October 1996*
*Revised 28 April 1997*
*Accepted 29 April 1997*

*Correspondence to:* CJA Punt, Department of Medical Oncology, University Hospital Nijmegen, PO Box 9101, 6500 HB Nijmegen, The Netherlands advanced colorectal and renal cell cancer were included in the study as these types of cancer are resistant to anthracyclins, with response rates of 7% (95% CI 1.9–17%) and 2.9% (95% CI 1.8–4.2%) respectively (Frytak et al, 1975; Yagoda et al, 1995). Although other mechanisms may play a role in their resistance to chemotherapy (Redmond et al, 1991; Chapman and Goldstein, 1995), tumour cells of both these types of cancer are known to express the *MDR1* gene and contain high levels of P-gp activity (Goldstein et al, 1989; Kanamaru et al, 1989; Lai et al, 1991; Kramer et al, 1993), and this may therefore play a key role in their resistance to anthracyclins. The aims of the present study were to investigate the toxicity, anti-tumour activity and pharmacokinetics of S9788 administered before and after infusion of doxorubicin in patients with advanced colorectal and renal cell cancer. A suboptimal dose of doxorubicin was chosen for this study (50 mg $m^{-2}$ once every 3 weeks) because of the possible potentiation of its side-effects by S9788.

### MATERIALS AND METHODS

### Inclusion criteria

Eligibility criteria included histological proof of advanced colorectal or renal cell cancer with documented progression within the last 2 months before entry into the study, measurable disease parameters, age between 18 and 75 years, Karnofsky performance status ≥80%, no radiotherapy, hormone therapy or immunotherapy during the last 2 weeks and no chemotherapy during the last 4 weeks before study entry. Normal values for Hb, platelets, WBC and serum electrolytes were required. Liver transaminases were allowed to be ≤ 2.5 times and bilirubin, amylase, creatinine and urea ≤ 1.25 times the upper normal values, and creatinine clearance ≥ 60 ml $min^{-1}$. Any history of significant cardiac arrhythmias, cardiac failure or recent myocardial infarction was not allowed, and patients were required to have normal cardiac ventricular ejection fraction (≥ 40%), no clinical signs of central nervous system metastasis, no concurrent use of other investigational or anti-neoplastic agents and no second malignancy. Written informed consent was obtained from all patients. Before initiation of this trial, institutional review board approval was obtained at each of the participating centres.

### Study design and drug administration

Patients were either initially treated with the combination of doxorubicin and S9788, or after documented progression of disease on treatment with doxorubicin alone. This decision was left to the investigators of the various institutions, as some centres considered treatment of patients with colorectal and renal cell cancer with single-agent doxorubicin not acceptable. S9788 (6-4-[2,2-di(4-fluorophenyl) ethylamino] 1-piperidinyl] N,N′-di,2-propenyl 1,3,5-triazine 2,4-diamine) was provided by IRIS (Courbevoie, France) in 10-ml vials at a concentration of 10 mg $ml^{-1}$ formulated in a bismethane sulphonate salt solution. The drug was diluted in either 250 ml (loading dose) or 1000 ml (2-h infusion) of dextrose 5% in water. A fixed loading dose of S9788 at 56 mg $m^{-2}$ administered over 30 min i.v. was followed by a 2-h infusion at different dose levels starting at 24 mg $m^{-2}$. Doxorubicin 50 mg $m^{-2}$ was administered over 5 min i.v. either alone or directly after the loading dose. A minimum of three patients were entered at each 2-h infusion dose level of S9788. No intra-patient dose escalation was allowed. Cycles were repeated every 3 weeks. All patients received antiemetic prophylaxis before doxorubicin infusion. Dose-limiting toxicity (DLT) was defined as any of the following events: (1) decrease ≥ 15% of ventricular ejection fraction; or (2) mucositis, cardiac, renal, hepatic, neurological or any major unexpected toxicity ≥ WHO grade 3. DLT occurring in two or more patients treated at the same dose level was considered as the clinical end point for this study.

### Study monitoring

A complete history, physical examination, performance status and laboratory studies [complete blood cell count with leucocyte differential (weekly), prothrombin and partial thromboplastin times, blood urea nitrogen, serum electrolytes, creatinine, calcium, phosphorus, liver transaminases, total bilirubin, amylase, alkaline phosphatase and urinalysis] were obtained at baseline and before each cycle. Ventricular ejection fraction, determined by either ultrasound or nuclear scanning, was planned for each patient at baseline and after completion of every two cycles. It was intended that all patients should have continuous cardiac telemetry and/or Holter recording for 24 h starting a minimum of 30 min before S9788 infusion or doxorubicin alone. Determination of the maximum corrected QT intervals (QTc max) was performed according to the method described by Bazett (1920). QTc values of ≤440 ms were considered as normal. Twelve-lead electrocardiograms were performed before and directly after each cycle. Patients were evaluated weekly for toxicity and every two cycles for response. Toxicity and response were scored according to WHO criteria. Cycles could only be repeated if granulocyte cell counts were ≥ 2000 × $10^6$ $l^{-1}$ and in the absence of grade ≥ 2 mucositis, renal, hepatic, neurological or other haematological toxicity at the time of retreatment.

### Pharmacokinetic analysis

Heparinized venous blood samples were collected before and at the end of the 30-min infusion of the loading dose of S9788 as well as before and 1 h, 2 h, 2.5 h and 24 h after the start of the 2-h maintenance infusion of S9788. Samples were quickly centrifuged and plasma was stored at –20°C until analysis. S9788 was quantitated by a specific high-performance liquid chromatographic method (HPLC) as described previously (Bakes et al, 1993) using a solid-phase extraction procedure and a reversed-phase HPLC (Hypersil ODS) with ultraviolet detection (230 nm). The mean precision and accuracy were 5.0% and 7.9%, respectively, over a range of 1–500 ng $ml^{-1}$ with a quantification limit of 1 ng $ml^{-1}$. S9788 plasma concentrations were modelled over time, using extended least squares regression on the computer program Micropharm (S. Vrien, LOGINSERM) version 4.0. The two-compartment model was chosen using Akaike's information criterion (Yamaoka et al, 1978). The total body clearance (Cl, h $l^{-1}$), distribution volume at steady state (*V*dss, I), distribution half-life ($t^1/_2\alpha$, min) and elimination half-life ($t^1/_2\beta$, h) were estimated by the model.

### Statistical methods

The correlation between dose of S9788 and QTc max was analysed using general linear models with the dose of S9788 as the main effect and the patient as the nested effect. The data from 63 cycles in 26 patients were analysed. The difference in non-cardiac toxicities between treatment with doxorubicin and doxorubicin plus S9788 was analysed ($\alpha = 0.05$) using the two-sided chi-square and the Fisher's exact tests.

© Cancer Research Campaign 1997

**Table 1** Dose escalation schedule of S9788

| Dose level of 2-h infusion of S9788 (mg m$^{-2}$) | Patients (*n*) | Total number of cycles |
|---|---|---|
| 24 | 4 | 12 |
| 48 | 6 | 13 |
| 72 | 9 | 29 |
| 96 | 10 | 26 |
| 120 | 5 | 9 |
| Total | 34 | 89 |

All patients received a loading dose of S9788 at 56 mg m$^{-2}$ i.v. in 30 min and doxorubicin at 50 mg m$^{-2}$ i.v. in 5 min prior to the 2-h infusion of S9788.

**Figure 1.** Correlation between QTc max values and S9788 dose levels of 24 mg m$^{-2}$ ($n = 4$), 48 mg m$^{-2}$ ($n = 12$), 72 mg m$^{-2}$ ($n = 19$), 96 mg m$^{-2}$ ($n = 22$), and 120 mg m$^{-2}$ ($n = 6$). S9788 at these dosages was administered as a 2-h infusion after a loading dose of S9788 at 56 mg m$^{-2}$ in 30 min followed by doxorubicin bolus infusion at 50 mg m$^{-2}$. The correlation was significant with $r = 0.38$, $P = 0.001$ (general linear models)

### RESULTS

### Patients characteristics

A total of 39 patients were entered into the study in eight participating institutions. One patient was considered ineligible because of pretreatment with doxorubicin at a cumulative dose of 490 mg m$^{-2}$ and a previous second malignancy. The number of assessable patients was therefore 38: 27 patients with colorectal cancer and 11 with renal cell cancer. The median age was 61 years (range 34–74) and median Karnofsky performance score was 90% (range 80–100%). The 28 patients pretreated with chemotherapy had received a median of two (1–3) regimens. The majority of these patients had received a 5-fluorouracil-containing regimen for colorectal cancer. Six of eleven patients with renal cell cancer had received prior immunotherapy.

### Treatment

Sixteen patients started treatment with doxorubicin alone at 50 mg m$^{-2}$ and received a median of two (1–5) cycles. Of these, 12 patients later received combined treatment with S9788 plus doxorubicin. The reasons that four patients did not receive the combined treatment were death due to progressive disease (1), multiple ventricular extrasystoles during doxorubicin treatment (1), patient refusal (1) and loss to follow-up (1). The remaining 22 patients started treatment with the combination of S9788 with doxorubicin.

The number of patients treated at the different dose levels of the 2-h infusion of S9788 is shown in Table 1. A total of 34 patients received a median of two cycles (1–8) of S9788. The planned dose for both drugs was respected in all patients but one, in whom the doxorubicin dose was reduced because of grade 3 stomatitis and grade 4 leucocytopenia. Although the clinical end point of the study was not reached, the study was terminated because of the occurrence of a torsade de pointe with syncope in a patient treated in another French phase I study at a S9788 dose that was lower than the doses that were used in our study (Terret et al, 1996).

### Cardiac toxicity

No major haemodynamic changes occurred in any patient during the study. No variation > 10% in the ventricular ejection fraction value was observed within individual patients. A total of 87 Holter registrations (73 of 24-h and 14 of 48-h) were obtained from 26 patients. On 19 Holter recordings of seven patients during administration of doxorubicin alone, no arrhythmias or prolongation of QTc max > 440 ms were observed. Asymptomatic arrhythmias, occurring after the start of S9788 infusion and disappearing within 18 h, were demonstrated on 21 out of 68 Holter recordings of 13 out of 34 patients receiving doxorubicin plus S9788. The following arrhythmias (frequency of occurrence) were seen: Mobitz type I (1), Mobitz type II (1), third-degree atrioventricular block (2), non-sustained ventricular tachycardia (3), supraventricular (8) and ventricular (4) extrasystoles, supraventricular tachycardias of less than 30 s duration (6). These arrhythmias occurred at all dose levels of S9788 and were not consistently present during every cycle in individual patients. In patients receiving doxorubicin plus S9788, the QTc max was ≤ 440 ms in one patient, > 440 and ≤ 600 ms in 20 patients, and > 600 ms in five patients, with a median value of 541 ms (range 404–685). QTc max occurred within 3 h after the start of S9788 infusion in 7 out of 26 patients, between 3 and 6 h in 16 out of 26 patients, and after 6 h in 3 out of 26 patients. There was a statistically significant corrrelation between the QTc max and the dose level of S9788 ($r = 0.38$, $P = 0.001$, Figure 1). An increase in the dose of S9788 of 24 mg m$^{-2}$ increased the QTc max with an average of 21.5 ms. There was no correlation between the QTc max and the occurrence of arrhythmias. No cumulative effect of S9788 on the QTc max was seen, but a cumulative effect on the occurrence of arrythmias could not be excluded.

### Non-cardiac toxicity

These consisted of alopecia, nausea, vomiting, stomatitis, and myelosuppression, as might be expected from treatment with doxorubicin. Compared with treatment with doxorubicin alone, treatment with the combination of doxorubicin plus S9788 caused a significant increase in the number of patients experiencing WHO grade 3–4 granulocytopenia (Table 2). No episode of febrile neutropenia occurred in any patient. A cumulative effect of doxorubicin as a cause for this toxicity was unlikely, as the first appearance of grade 3–4 granulocytopenia occurred during the

© Cancer Research Campaign 1997

**Table 2** Non-cardiac toxicities

| Toxicities | WHO grade | Doxorubicin | Doxorubicin plus S9788 | P-value overall/grade 3–4 |
|---|---|---|---|---|
| Nausea | 0 | 10 (62%) | 10 (29%) | |
| | 1/2 | 6 (38%) | 24 (71%) | |
| | 3/4 | 0 | 0 | 0.06/1.0 |
| Vomiting | 0 | 12 (80%) | 17 (50%) | |
| | 1/2 | 2 (13%) | 14 (41%) | |
| | 3/4 | 1 (7%) | 3 (9%) | 0.13/1.0 |
| Stomatitis | 0 | 16 (100%) | 28 (82%) | |
| | 1/2 | 0 | 3 (9%) | |
| | 3/4 | 0 | 3 (9%) | 0.20/0.61 |
| Anaemia | 0 | 12 (75%) | 14 (47%) | |
| | 1/2 | 4 (25%) | 13 (43%) | |
| | 3/4 | 0 | 3 (10%) | 0.10/0.53 |
| Leucopenia | 0 | 4 (25%) | 4 (14%) | |
| | 1/2 | 10 (62%) | 13 (45%) | |
| | 3/4 | 2 (13%) | 12 (41%) | 0.09/0.09 |
| Granulocytopenia | 0 | 5 (31%) | 6 (22%) | |
| | 1/2 | 8 (50%) | 6 (22%) | |
| | 3/4 | 3 (19%) | 15 (56%) | 0.05/0.04 |
| Thrombocytopenia | 0 | 15 (94%) | 33 (97%) | |
| | 1/2 | 1 (6%) | 0 | |
| | 3/4 | 0 | 1 (3%) | 0.27/1.0 |

The incidence of overall toxicity (three categories: grade 0, grade 1–2, grade 3–4) and of grade 3–4 toxicity in patients treated with doxorubicin vs doxorubicin plus S9788 was analysed two-sided ($\alpha = 0.05$) with the chi-square test (overall toxicity) and the exact test (grade 3–4 toxicity). A significant difference was noted in the incidence of grade 3–4 leucocytopenia and granulocytopenia. Numbers are patients.

**Figure 2.** S9788 plasma concentration profile of a patient receiving S9788 at a 30 min loading dose of 56 mg $m^{-2}$ followed by a 5-min bolus infusion of doxorubicin and a S9788 2-h maintenance dose of 72 mg $m^{-2}$. *x*-axis, time from start infusion of loading dose of S9788; *y*-axis, S9788 concentration (μM)

first cycle of doxorubicin plus S9788 in 76% of patients, and 5 out of 12 patients with grade 3–4 leucocytopenia and 8 out of 15 patients with grade 3–4 granulocytopenia were not initially treated with cycles of doxorubicin alone. No statistically significant differences were found between treatment with doxorubicin and with doxorubicin plus S9788 when overall toxicity was observed (Table 2). When the incidence of toxicity was compared with the number of cycles with and without S9788, a statistically significant increase was also found in the incidence of nausea (grade 1–2) and vomiting (grade 1–3) in cycles with doxorubicin plus S9788. Nausea increased from an incidence of 6 out of 38 of cycles with doxorubicin alone (16%) to 43 out of 89 of cycles with doxorubicin plus S9788 (48%) ($P = 0.001$), and vomiting from 3 out of 38 cycles (8%) to 30 out of 89 cycles (34%) ($P = 0.005$). No significant differences occurred in the incidence or grade of diarrhoea, hepatic or renal toxicity (data not shown).

### S9788 pharmacokinetics

A total of eight patients were included in the pharmacokinetic study. All these patients received a loading dose of S9788 of 56 mg $m^{-2}$ followed by doxorubicin infusion. Thereafter, three patients received a 2-h infusion of S9788 at 24 mg $m^{-2}$, one patient at 48 mg $m^{-2}$, two patients at 72 mg $m^{-2}$ and two patients at 96 mg $m^{-2}$. A typical plasma concentration profile of S9788 over time is presented in Figure 2. The mean ± standard deviation (s.d.) of the maximum plasma concentration reached at the end of the 56 mg $m^{-2}$ loading dose was 1.31 ± 0.41 μM (range 0.73–2.00). The maximum values for S9788 concentration during the 2-h maintenance infusion increased with the administration dose from 0.38 ± 0.11 μM up to 1.05 ± 0.50 μM for 24 and 96 mg $m^{-2}$ respectively. The mean ± s.d. pharmacokinetic parameters were as follows: Cl = 47 ± 18 l $h^{-1}$, *V*dss = 669 ± 247 l, $t^1/_2\alpha = 7 \pm 5$ min, $t^1/_2\beta$ 14 ± 10 h.

### Clinical response

Of the 34 patients who received doxorubicin plus S9788, 29 were evaluable for response. Of these, one patient with colorectal cancer had a partial response of 6 months' duration after disease progression on two cycles of doxorubicin alone. Three patients (one colorectal and two renal cell cancer) treated with doxorubicin plus S9788 had stable disease for 3, 4 and 7 months respectively. The remaining 25 patients had progressive disease.

### DISCUSSION

Clinical studies with MDR-modulating agents have shown disappointing results so far, mainly because of toxicities occurring at doses that were needed to achieve relevant plasma concentrations of the MDR modulator. Effective MDR reversal by S9788 in vitro has been observed, beginning at concentrations of 0.25 μM (Soudon et al, 1995). Therefore, this study shows that, using this schedule, effective concentrations of S9788 can be reached at non-toxic doses in patients. The high total body clearance and short initial half-life of S9788 that we found support the rationale for prolonged infusion over bolus infusion of S9788, as was suggested by others (Perez et al, 1993; Julia et al, 1994; Soudon et al, 1995). Compared with treatment with doxorubicin alone, patients treated with doxorubicin plus S9788 experienced a significant increase in the occurrence of WHO grade 3–4 granulocytopenia, but not in the occurrence or severity of other toxicities. When toxicities were compared on a per cycle basis, there was also an increase in the occurrence of nausea and vomiting. A cumulative effect of doxorubicin is unlikely as these toxicities mainly occurred during the first cycle of doxorubicin plus S9788, and occurred equally in patients initially treated with and without cycles of doxorubicin without S9788. A pharmacokinetic interaction between S9788 and doxorubicin, as has been shown for instance for verapamil and epirubicin (Scheithauer et al, 1993) and cyclosporine and doxorubicin (Bartlett et al, 1994), can be excluded as the interference of S9788 with the pharmacokinetics of doxorubicin has been investigated by the intra-individual comparisons of the pharmacokinetics parameters of doxorubicin obtained during two different cycles of doxorubicin treatment without or with S9788 administration (de Valeriola et al, 1997). In this study, S9788 was not shown to interfere with doxorubicin pharmacokinetics.

The most common doxorubicin-induced cardiotoxicity is a cumulative dose-related myocardial cell damage that may result in congestive heart failure. Acute electrocardiographical changes and/or arrhythmias during and shortly after administration have also been described and consist primarily of reversible non-specific ST–T segment changes, sinus tachycardia, premature atrial and ventricular contractions, and decrease in voltage (Tokaz and Von Hoff, 1984). The incidence of these abnormalities ranges from 0 to 41%. Prolongation of the QTc interval associated with arrhythmias has been described during anthracycline therapy in children and occurred more frequently in patients who received high cumulative doses (Bender et al, 1984; Schwartz et al, 1993). In this study, we found no electrocardiographical changes or arrhythmias in patients treated with doxorubicin alone, except for one patient who experienced multiple ventricular extrasystoles. However, in the 26 patients of whom Holter recordings were available during treatment with the combination of doxorubicin and S9788, a prolongation of QTc max and cardiac arrhythmias occurred in 25 out of 26 and 13 out of 26 patients respectively. Although it has been demonstrated that Holter recordings of healthy subjects show arrhythmias in a significant percentage (Stinson et al, 1995), a causal relationship between S9788 and these arrythmias seemed probable as they occurred after the start of S9788 infusion and disappeared within 18 h. A correlation was established between the dose of S9788 and the prolongation of QTc max, although there was a high variation between individuals. There was no correlation between the dose of S9788 and the occurrence of arrhythmias. However, it should be noted that this study did not have the appropriate design to establish these correlations for the following reasons: QTc max is known to vary between and within individuals, and the QTc dispersion (i.e. the distribution of repolarization on the heart) rather than the absolute value of QTc max may be considered as a risk factor for the occurrence of arrhythmias (Surawicz and Knoebel, 1984). Moreover, in our study patients were not randomized for the different S9788 dose levels. In contrast, Terret et al (1996) found no correlation between QTc lengthening and dose of S9788 in other phase I studies. This might be explained by the above-mentioned reasons, and/or by the fact that other doses and schedules of S9788 were used. Although a predictive value of QTc lengthening for the occurrence of severe arrhythmias has never been established for values < 600 ms (Surawicz and Knoebel, 1984), the study was terminated after the occurrence of severe cardiac arrhythmias (torsade de pointe with syncope) in another ongoing study with S9788 given over 6 h (Terret et al, 1996). Such a risk would preclude the routine use of S9788.

The clinical activity of treatment with doxorubicin plus S9788 was limited to one partial response in a patient with colorectal cancer, but a causative role of S9788 was obvious as this patient had disease progression during prior treatment with doxorubicin alone. It should be noted that a suboptimal dose of doxorubicin was chosen for safety reasons.

In conclusion, we have safely administered a combination treatment of doxorubicin and S9788 to 39 patients, and with the doses used relevant concentrations of S9788 were achieved. However, because of the unpredictable occurrence of cardiac arrhythmias, the company has decided to withdraw the drug S9788 from further clinical development.

### ACKNOWLEDGEMENTS

The statistical analysis was performed by W Doesburg, Department of Medical Statistics, University of Nijmegen, The Netherlands, and is greatly appreciated. The study was supported by IRIS Courbevoie, France.

### REFERENCES

Awada A, Pagani O, Piccart M, Leyvraz S, Cerny T, Sessa C, Cavalli F, Coquoz D, Marinus W and Gerard B (1993) Phase I clinical and pharmacokinetic trials of S9788 alone and in combination with adriamycin. (Abstract) *Proc Am Assoc Cancer Res* **34**: 213

Bakes DM, Turner ND, Gordon BH, Hiley MP and Walther B (1993) Method for the analysis of S9788, a drug to reverse resistance to anticancer agents, in animal plasms and human plasma and serum by high-performance liquid chromatography with ultraviolet detection. *J Chromatogr* **615**: 117–126

Bartlett NL, Lum BL, Fisher GA, Brophy NA, Ehsan MN, Halsey J and Sikic BI (1994) Phase I trial of doxorubicin with cyclosporine as a modulator of multidrug resistance. *J Clin Oncol* **12**: 835–842

Bazett HC (1920) An analysis of the time-relations of electrocardiograms. *Heart* **7**: 353

Bender KS, Shematek JP, Leventhal BG and ET AL (1984) QT interval prolongation associated with anthracycline cardiotoxicity. *J Pediatr* **105**: 442–444

Chapman AE and Goldstein LJ (1995) Multiple drug resistance: Biologic basis and clinical significance in renal-cell carcinoma. *Semin Oncol* **22**: 17–28

Clavel M, Sarkany M, Catimel G, Dumortier A, Guastalla JP, Ardiet C, Lucas C and Bizzari JP (1993) Phase I trial of S9788 in combination with adriamycin as a new multidrug resistance reverser in solid tumors. (Abstract) *Proc Am Assoc Cancer Res* **34**: 231

Cros S, Guilbaud N, Berlion M, Dunn T, Regnier G, Dhainaut A, Atassi G and Bizzari JP (1992) In vivo evidence of complete circumvention of vincristine resistance by a new triazinoaminopiperidine derivate S9788 in P388/VCR leukemia model. *Cancer Chemother Pharmacol* **30**: 491–494

De Valeriola D, Brassinne C, Lucas S, Gerard B, Tueni E, Awada A, Parmentier N, Bleiberg H, Piccart M (1997) Lack of interference of S9788 with the

© Cancer Research Campaign 1997

pharmacokinetics of doxorubicin. *Cancer Chemotherapy Pharmacology* (in press)

Efferth T, Dunn TA, Berlion M, Langenbahn H, Pommerenke EW and Volm M (1993) Reversal of inherent multidrug-resistance in primary human renal cell carcinoma cell cultures by S9788. *Anticancer Res* **13**: 905–908

Frytak S, Mortel CG, Schutt AJ, Hahn RG and Reitemeier RJ (1975) Adriamycin (NSC-123127) therapy for advanced gastrointestinal cancer. *Cancer Chemother Rep* **59**: 405–409

Goldstein LJ, Galski H, Fojo A, Willingham M, Lai SL, Gazdar A, Pirker R, Green A, Crist W, Brodeur GM, Lieber M, Cossman J, Gottesman MM and Pastan I (1989) Expression of a multidrug resistance gene in human cancers. *J Natl Cancer Inst* **81**: 116–124

Goncalves E, Sarkany M, Da Costa L, Fadel E, Theodore C, Lucas C, Giroux B and Armand JP (1995) S9788, new multidrug resistance reversing agent: phase I study of a 6 hour continuous infusion in combination with doxorubicin in patients with refractory cancer. (Abstract) *Proc Am Soc Clin Oncol* **14**: 182

Hill BT, Van Der Graaf WTA, Hosking LK, De Vries EGE, Mulder NH and Whelan RDH (1993) Evaluation of S9788 as a potential modulator of drug resistance against human tumour sublines expressing differing resistance mechanisms in vitro. *Int J Cancer* **55**: 330–337

Huet S, Chapey C and Robert J (1993) Reversal of multidrug resistance by a new lipophilic cationic molecule, S9788. Comparison with 11 other MDR-modulating agents in a model of doxorubicin-resistant rat glioblastoma cells. *Eur J Cancer* **29A**: 1377–1383

Julia AM, Roche H, Berlion M, Milano G, Robert J, Bizzari JP and Canal P (1994) Multidrug resistance circumvention by a new triazinoaminopiperidine derivate S9788 in vitro: definition of the optimal schedule and comparison with verapamil. *Br J Cancer* **69**: 868–874

Kanamaru H, Kakehi Y, Yoshida O, Nakanishi S, Pastan I and Gottesman MM (1989) MDR1 RNA levels in human renal cell carcinomas: correlation with grade and prediction of reversal of doxorubicin resistance by quinidine in tumor explants. *J Natl Cancer Inst* **81**: 844–849

Kramer R, Weber TK, Morse B, Staniunas R, Steele G, Jr and Summerhayes IC (1993) Constitutive expression of multidrug resistance in human colorectal tumours and cell lines. *Br J Cancer* **67**: 959–968

Lai GM, Chen YN, Mickley LA, Fojo AT and Bates SE (1991) P-glycoprotein expression and schedule dependence of adriamycin cytotoxicity in human colon carcinoma cell lines. *Int J Cancer* **49**: 696–703

Leonce S, Pierre A, Anstett M, Perez V, Genton A, Bizzari JP and Atassi G (1992) Effects of a new triazinoaminopiperidine derivate on adriamycin accumulation and retention in cells displaying P-glycoprotein-mediated multidrug resistance. *Biochem Pharmacol* **44**: 1707–1715

Merlin JM, Guerci A, Marchal S, Missoum N, Ramacci C, Humbert JC, Tsuruo T and Guerci O (1994) Comparative evaluation of S9788, verapamil, and cyclosporin A in K562 human leukemia cell lines and in P-glycoprotein-expressing samples from patients with hematologic malignancies. *Blood* **84**: 262–269

Pennock GD, Dalton WS, Roeske WR, Appleton CP, Mosley K, Plezia P, Miller TP and Salmon SE (1991) Systemic toxic effects associated with high-dose verapamil infusion and chemotherapy administration. *J Natl Cancer Inst* **83**: 105–110

Perez V, Pierre A, Leonce S, Anstett M and Atassi G (1993) Effect of duration of exposure to S9788, cyclosporin A or verapamil on sensitivity of multidrug resistant cells to vincristine or doxorubicin. *Anticancer Res* **13**: 985–990

Pierre A, Dunn TA, Kraus-Berthier L, Leonce S, Saint-Dizier D, Regnier G, Dhainaut A, Berlion M, Bizzari JP and Atassi G (1992). In vitro and in vivo circumvention of multidrug resistance by Servier 9788, a novel triazinoaminopiperidine derivative. *Invest New Drugs* **10**: 137–148

Raderer M and Scheithauer W (1993) Clinical trials of agents that reverse multidrug resistance. A literature review. *Cancer* **72**: 3553–3563

Redmond SMS, Joncourt F, Buser K, Ziemiecki A, Altermatt HJ, Fey M, Margison G and Cerny T (1991) Assessment of P-glycoprotein, glutathione-based detoxifying enzymes and O6-alkylguanine-DNA alkyltransferase as potential indicators of constitutive drug resistance in human colorectal tumors. *Cancer Res* **51**: 2092–2097

Roninson IB (1992) The role of the MDR1 (P-glycoprotein) gene in multidrug resistance in vitro and in vivo. *Biochem Pharmacol* **43**: 95–102

Scheithauer W, Schenk T and Czejka M (1993) Pharmacokinetic interaction between epirubicin and the multidrug resistance reverting agent D-verapamil. *Br J Cancer* **68**: 8–9

Schwartz CL, Hobbie WL, Truesdell S, Constine LC and Clark EB (1993) Corrected QT interval prolongation in anthracycline-treated survivors of childhood cancer. *J Clin Oncol* **11**: 1906–1910

Sebille S, Morjani H, Poullain MG and Manfait M (1994) Effect of S9788, cyclosporin A and verapamil on intracellular distribution of THP-doxorubicin in multidrug-resistant K562 tumor cells, as studied by laser confocal microspectrofluorometry. *Anticancer Res* **14**: 2389–2394

Soudon J, Berlion M, Lucas C, Haddad P, Bizzari JP and Calvo F (1995) In vitro activity of S 9788 on a multidrug-resistant leukemic cell line and on normal hematopoietic cells – reversal of multidrug resistance by sera from phase I treated patients. *Cancer Chemother Pharmacol* **36**: 195–203

Stinson JC, Pears JS, Williams AJ and Campbell RWF (1995) Use of 24 th ambulatory ECG recordings in the assessment of new chemical entities in healthy volunteers. *Br J Clin Pharmacol* **39**: 651–656

Surawicz B and Knoebel SB (1984) Long QT: good, bad or indifferent? *JACC* **4**: 398–413

Terret C, Le Cesne A, Lagarde N, Di Palma M, Goncalves E, N'Dom P, Yataghene Y, Funck-Brentano C, N'Guyen JP, Marino JP, Besse B, Armand JP, Le Chevalier T, Belpomme D, Misset JL, D'Agay L, Berger E, Sarkany M, Giroux B (1996) S9788 a multidrug resistance (MDR) reversing agent: French phase I studies with a 6 hour continuous infusion schedule in combination with chemotherapy in patients with refractory cancer. (Abstract) *Proc Am Assoc Cancer Res* **37**: 165–166

Tokaz LK and Von Hoff DD (1984) The cardiotoxicity of anticancer agents. In: *Toxicity of Chemotherapy*, Perry MC and Yarbro JW (eds) pp. 199–226. Grune & Stratton: Orlando, FL, USA.

Yagoda A, Abirached B and Petrylak D (1995) Chemotherapy for advanced renal-cell carcinoma: 1983–1993. *Semin Oncol* **22**: 42–60

Yamaoka K, Nakagawa T and Uno T (1978) Application of Akaike's Information Criterion (AIC) in the evaluation of linear pharmacokinetic equations. *J Pharmacokinet Biopharm* **6**: 165

© Cancer Research Campaign 1997


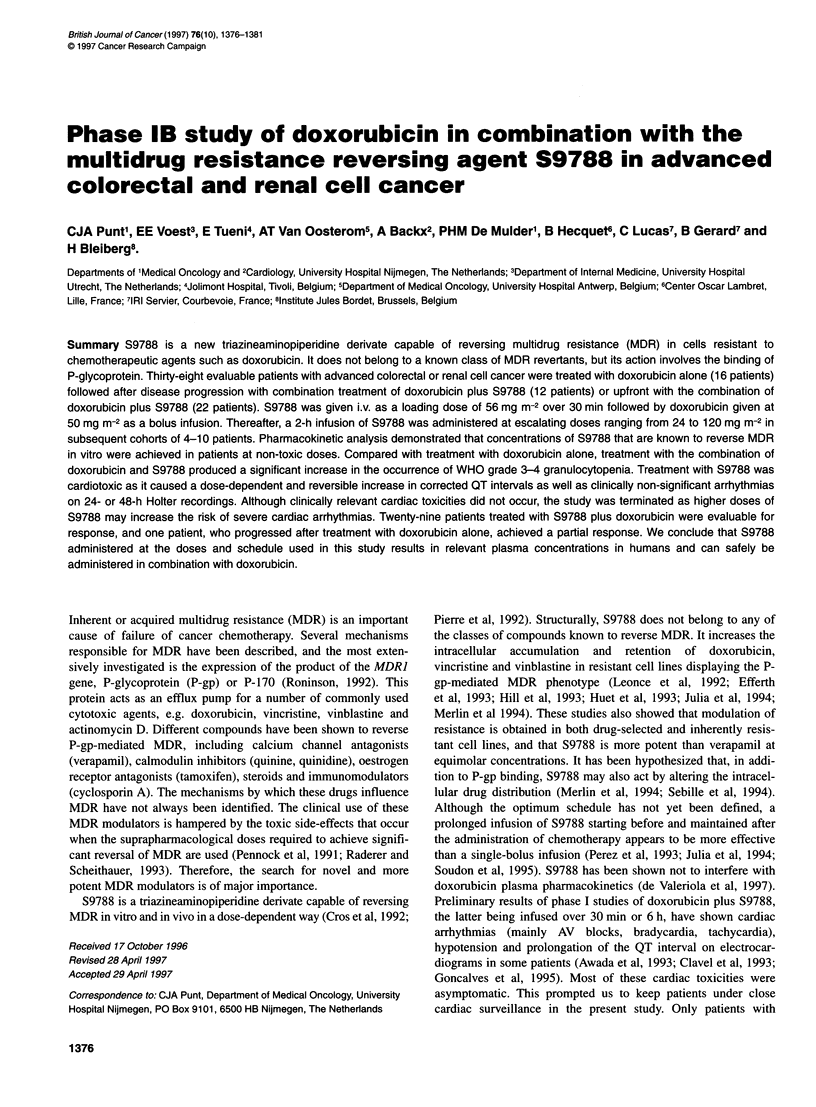

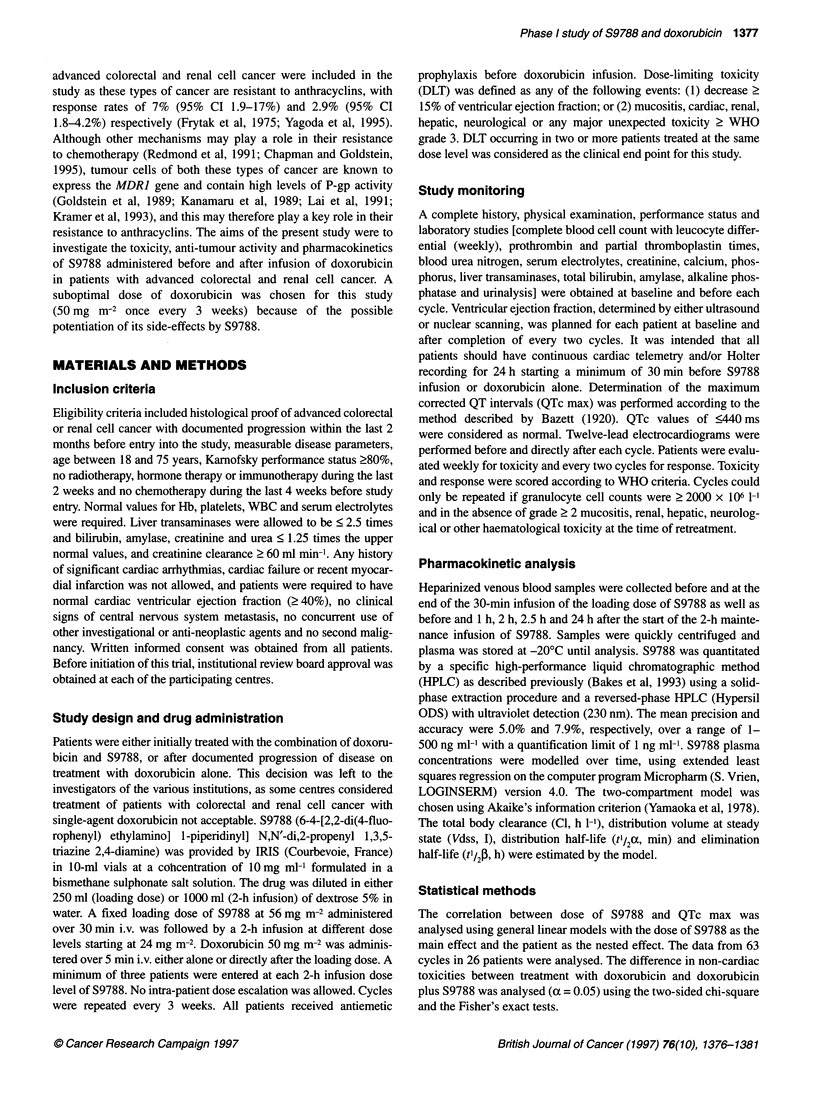

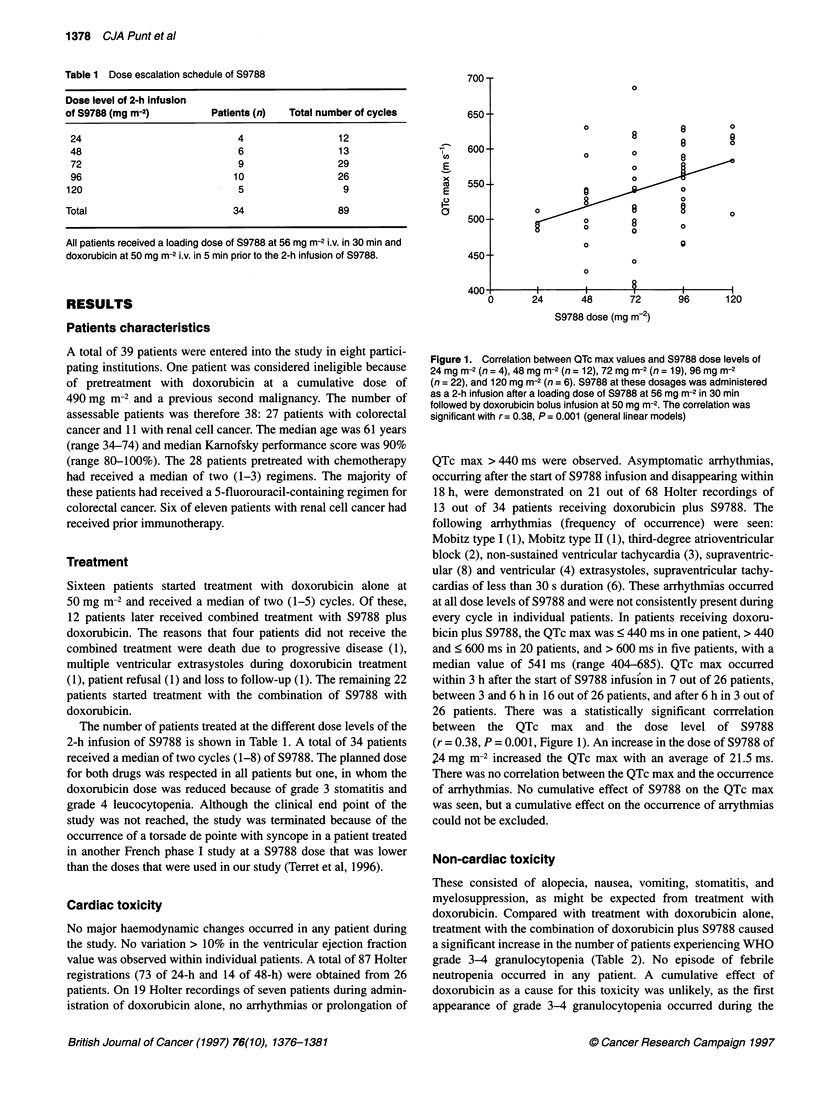

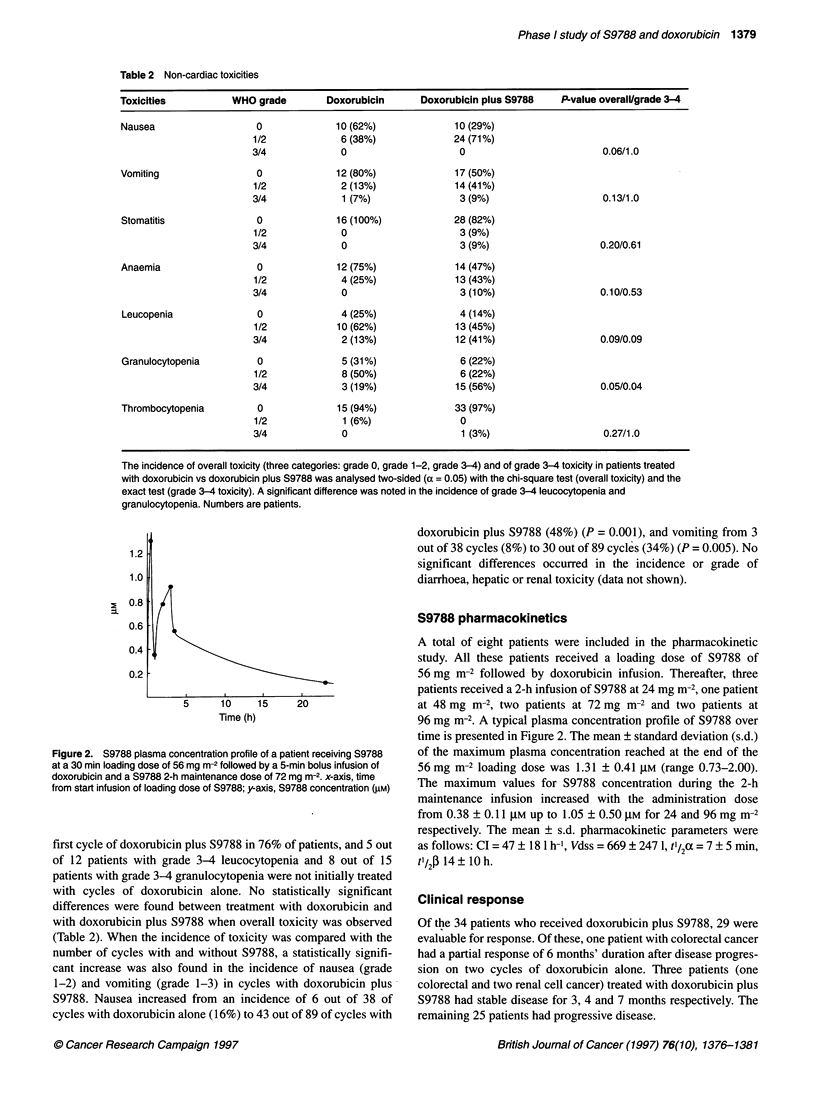

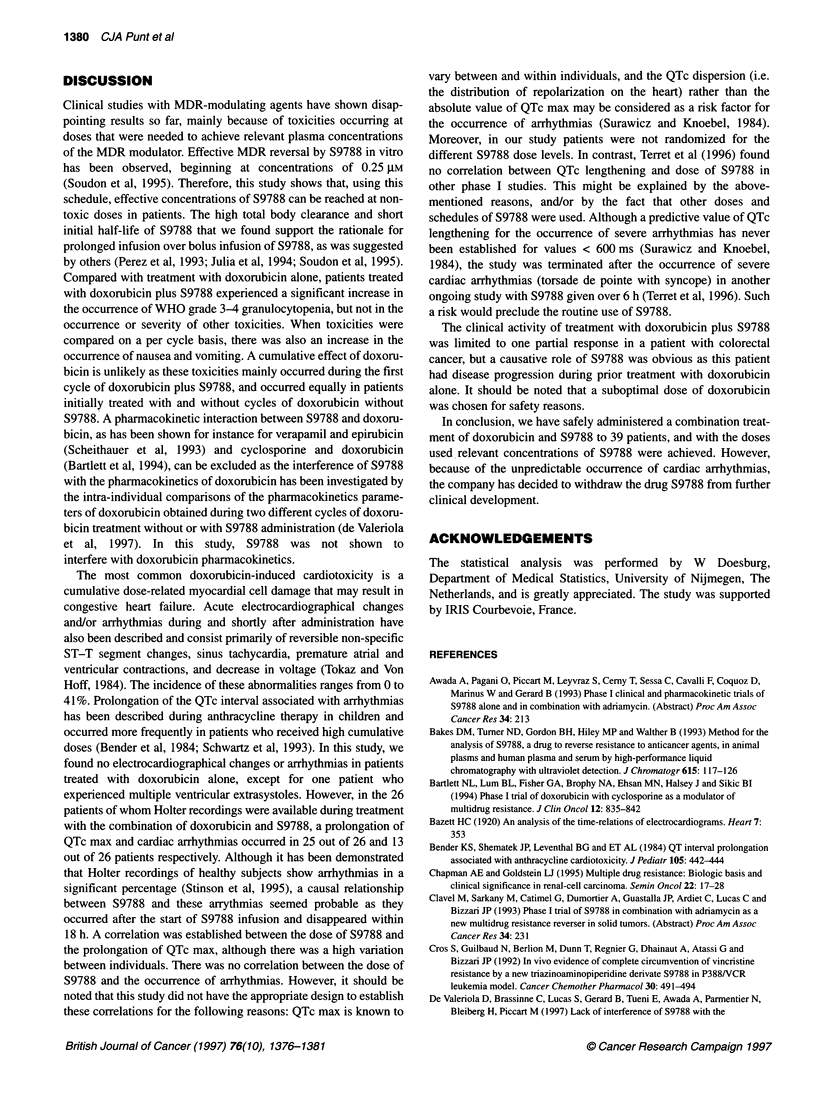

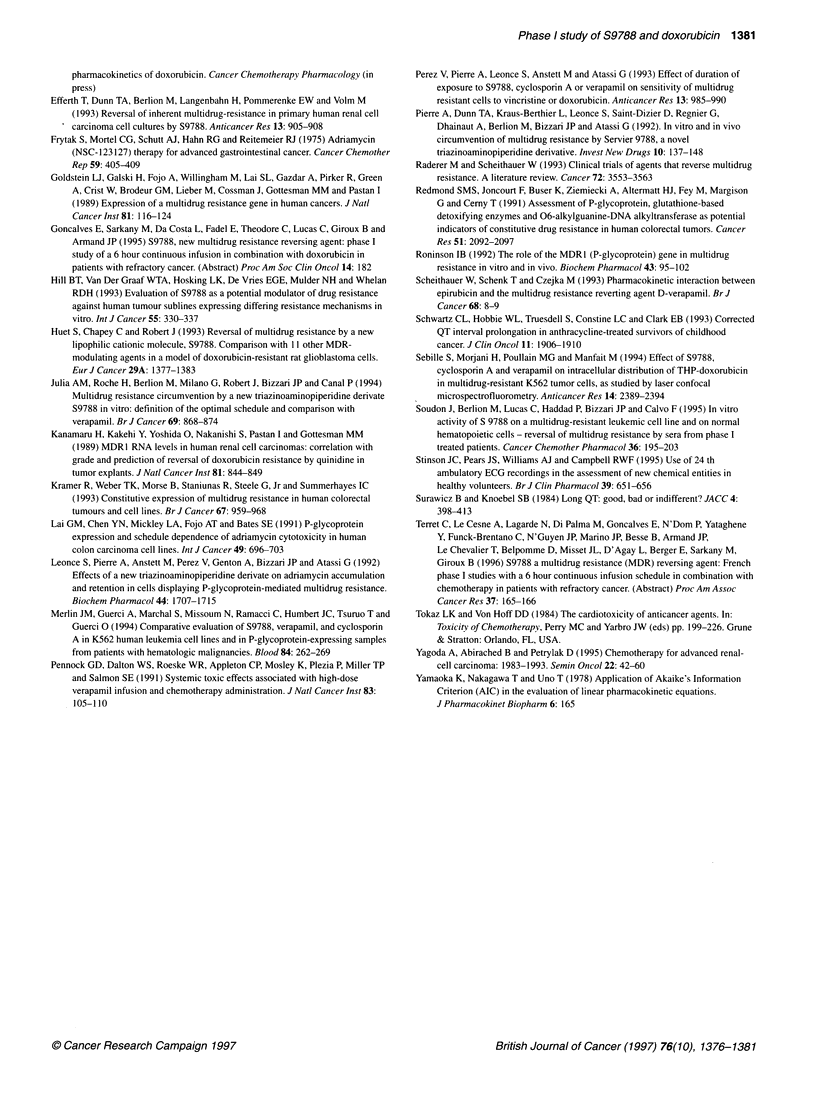


## References

[OCR_00623] Bakes D. M., Turner N. D., Gordon B. H., Hiley M. P., Walther B., Lucas C. (1993). Method for the analysis of S9788, a drug to reverse resistance to anticancer agents, in animal plasma and human plasma and serum by high-performance liquid chromatography with ultraviolet detection.. J Chromatogr.

[OCR_00630] Bartlett N. L., Lum B. L., Fisher G. A., Brophy N. A., Ehsan M. N., Halsey J., Sikic B. I. (1994). Phase I trial of doxorubicin with cyclosporine as a modulator of multidrug resistance.. J Clin Oncol.

[OCR_00639] Bender K. S., Shematek J. P., Leventhal B. G., Kan J. S. (1984). QT interval prolongation associated with anthracycline cardiotoxicity.. J Pediatr.

[OCR_00643] Chapman A. E., Goldstein L. J. (1995). Multiple drug resistance: biologic basis and clinical significance in renal-cell carcinoma.. Semin Oncol.

[OCR_00653] Cros S., Guilbaud N., Berlion M., Dunn T., Regnier G., Dhainaut A., Atassi G., Bizzari J. P. (1992). In vivo evidence of complete circumvention of vincristine resistance by a new triazinoaminopiperidine derivative S 9788 in P388/VCR leukemia model.. Cancer Chemother Pharmacol.

[OCR_00670] Efferth T., Dunn T. A., Berlion M., Langenbahn H., Pommerenke E. W., Volm M. (1993). Reversal of inherent multidrug-resistance in primary human renal cell carcinoma cell cultures by S 9788.. Anticancer Res.

[OCR_00675] Frytak S., Moertel C. G., Schutt A. J., Hahn R. G., Reitemeier R. J. (1975). Adriamycin (NSC-123127) therapy for advanced gastrointestinal cancer.. Cancer Chemother Rep.

[OCR_00680] Goldstein L. J., Galski H., Fojo A., Willingham M., Lai S. L., Gazdar A., Pirker R., Green A., Crist W., Brodeur G. M. (1989). Expression of a multidrug resistance gene in human cancers.. J Natl Cancer Inst.

[OCR_00693] Hill B. T., van der Graaf W. T., Hosking L. K., de Vries E. G., Mulder N. H., Whelan R. D. (1993). Evaluation of S9788 as a potential modulator of drug resistance against human tumour sublines expressing differing resistance mechanisms in vitro.. Int J Cancer.

[OCR_00699] Huet S., Chapey C., Robert J. (1993). Reversal of multidrug resistance by a new lipophilic cationic molecule, S9788. Comparison with 11 other MDR-modulating agents in a model of doxorubicin-resistant rat glioblastoma cells.. Eur J Cancer.

[OCR_00706] Julia A. M., Roché H., Berlion M., Lucas C., Milano G., Robert J., Bizzari J. P., Canal P. (1994). Multidrug resistance circumvention by a new triazinoaminopiperidine derivative S9788 in vitro: definition of the optimal schedule and comparison with verapamil.. Br J Cancer.

[OCR_00712] Kanamaru H., Kakehi Y., Yoshida O., Nakanishi S., Pastan I., Gottesman M. M. (1989). MDR1 RNA levels in human renal cell carcinomas: correlation with grade and prediction of reversal of doxorubicin resistance by quinidine in tumor explants.. J Natl Cancer Inst.

[OCR_00718] Kramer R., Weber T. K., Morse B., Arceci R., Staniunas R., Steele G., Summerhayes I. C. (1993). Constitutive expression of multidrug resistance in human colorectal tumours and cell lines.. Br J Cancer.

[OCR_00723] Lai G. M., Chen Y. N., Mickley L. A., Fojo A. T., Bates S. E. (1991). P-glycoprotein expression and schedule dependence of adriamycin cytotoxicity in human colon carcinoma cell lines.. Int J Cancer.

[OCR_00728] Léonce S., Pierré A., Anstett M., Pérez V., Genton A., Bizzari J. P., Atassi G. (1992). Effects of a new triazinoaminopiperidine derivative on adriamycin accumulation and retention in cells displaying P-glycoprotein-mediated multidrug resistance.. Biochem Pharmacol.

[OCR_00734] Merlin J. L., Guerci A., Marchal S., Missoum N., Ramacci C., Humbert J. C., Tsuruo T., Guerci O. (1994). Comparative evaluation of S9788, verapamil, and cyclosporine A in K562 human leukemia cell lines and in P-glycoprotein-expressing samples from patients with hematologic malignancies.. Blood.

[OCR_00740] Pennock G. D., Dalton W. S., Roeske W. R., Appleton C. P., Mosley K., Plezia P., Miller T. P., Salmon S. E. (1991). Systemic toxic effects associated with high-dose verapamil infusion and chemotherapy administration.. J Natl Cancer Inst.

[OCR_00751] Pierré A., Dunn T. A., Kraus-Berthier L., Léonce S., Saint-Dizier D., Régnier G., Dhainaut A., Berlion M., Bizzari J. P., Atassi G. (1992). In vitro and in vivo circumvention of multidrug resistance by Servier 9788, a novel triazinoaminopiperidine derivative.. Invest New Drugs.

[OCR_00747] Pérez V., Pierré A., Léonce S., Anstett M., Atassi G. (1993). Effect of duration of exposure to S9788, cyclosporin A or verapamil on sensitivity of multidrug resistant cells to vincristine or doxorubicin.. Anticancer Res.

[OCR_00758] Raderer M., Scheithauer W. (1993). Clinical trials of agents that reverse multidrug resistance. A literature review.. Cancer.

[OCR_00762] Redmond S. M., Joncourt F., Buser K., Ziemiecki A., Altermatt H. J., Fey M., Margison G., Cerny T. (1991). Assessment of P-glycoprotein, glutathione-based detoxifying enzymes and O6-alkylguanine-DNA alkyltransferase as potential indicators of constitutive drug resistance in human colorectal tumors.. Cancer Res.

[OCR_00770] Roninson I. B. (1992). The role of the MDR1 (P-glycoprotein) gene in multidrug resistance in vitro and in vivo.. Biochem Pharmacol.

[OCR_00774] Scheithauer W., Schenk T., Czejka M. (1993). Pharmacokinetic interaction between epirubicin and the multidrug resistance reverting agent D-verapamil.. Br J Cancer.

[OCR_00779] Schwartz C. L., Hobbie W. L., Truesdell S., Constine L. C., Clark E. B. (1993). Corrected QT interval prolongation in anthracycline-treated survivors of childhood cancer.. J Clin Oncol.

[OCR_00784] Sebille S., Morjani H., Poullain M. G., Manfait M. (1994). Effect of S9788, cyclosporin A and verapamil on intracellular distribution of THP-doxorubicin in multidrug-resistant K562 tumor cells, as studied by laser confocal microspectrofluorometry.. Anticancer Res.

[OCR_00790] Soudon J., Berlion M., Lucas C., Haddad P., Bizzari J. P., Calvo F. (1995). In vitro activity of S 9788 on a multidrug-resistant leukemic cell line and on normal hematopoietic cells-reversal of multidrug resistance by sera from phase I-treated patients.. Cancer Chemother Pharmacol.

[OCR_00796] Stinson J. C., Pears J. S., Williams A. J., Campbell R. W. (1995). Use of 24 h ambulatory ECG recordings in the assessment of new chemical entities in healthy volunteers.. Br J Clin Pharmacol.

[OCR_00801] Surawicz B., Knoebel S. B. (1984). Long QT: good, bad or indifferent?. J Am Coll Cardiol.

[OCR_00821] Yagoda A., Abi-Rached B., Petrylak D. (1995). Chemotherapy for advanced renal-cell carcinoma: 1983-1993.. Semin Oncol.

[OCR_00825] Yamaoka K., Nakagawa T., Uno T. (1978). Application of Akaike's information criterion (AIC) in the evaluation of linear pharmacokinetic equations.. J Pharmacokinet Biopharm.

